# Metabolomic Profiling and Anti-*Helicobacter pylori* Activity of *Caulerpa lentillifera* (Sea Grape) Extract

**DOI:** 10.3390/md23070282

**Published:** 2025-07-07

**Authors:** Chananchida Thacharoen, Thisirak Inkaewwong, Watthanachai Jumpathong, Pornchai Kaewsapsak, Thiravat Rattanapot, Tippapha Pisithkul

**Affiliations:** 1 Program in Biotechnology, Faculty of Science, Maejo University, Chiang Mai 50290, Thailand; 2 Program in Chemical Sciences, Chulabhorn Graduate Institute, Bangkok 10210, Thailand; watthanachai@cgi.ac.th; 3 Chulabhorn Royal Academy, Bangkok 10210, Thailand; 4 Center of Excellence in Systems Microbiology (CESM), Department of Biochemistry, Faculty of Medicine, Chulalongkorn University, Bangkok 10330, Thailand; pkaewsap@gmail.com; 5 Center of Excellence in Agriculture Innovation for Graduate Entrepreneur, Maejo University, Chiang Mai 50290, Thailand; 6 Unit of Excellence in Modern Plant Biotechnology, Faculty of Science, Maejo University, Chiang Mai 50290, Thailand

**Keywords:** *Caulerpa lentillifera*, sea grape, chemical composition, metabolomics, *Helicobacter pylori*

## Abstract

*Helicobacter pylori* is a gastric pathogen implicated in peptic ulcer disease and gastric cancer. The increasing prevalence of antibiotic-resistant strains underscores the urgent need for alternative therapeutic strategies. In this study, we investigated the chemical composition and antibacterial activity of an aqueous extract from *Caulerpa lentillifera* (sea grape), a farm-cultivated edible green seaweed collected from Krabi Province, Thailand. Ultra-high-performance liquid chromatography–tandem mass spectrometry (UHPLC-MS/MS) revealed that the extract was enriched in bioactive nucleosides and phenolic compounds. In vitro assays demonstrated dose-dependent inhibition of *H. pylori* growth following exposure to sea grape extract. Furthermore, untargeted intracellular metabolomic profiling of *H. pylori* cells treated with the extract uncovered significant perturbations in central carbon and nitrogen metabolism, including pathways associated with the tricarboxylic acid (TCA) cycle, one-carbon metabolism, and alanine, aspartate, and glutamate metabolism. Pyrimidine biosynthesis was selectively upregulated, indicating a potential stress-induced shift toward nucleotide salvage and DNA repair. Of particular note, succinate levels were markedly reduced despite accumulation of other TCA intermediates, suggesting disruption of electron transport-linked respiration. These findings suggest that bioactive metabolites from *C. lentillifera* impair essential metabolic processes in *H. pylori*, highlighting its potential as a natural source of antimicrobial agents targeting bacterial physiology.

## 1. Introduction

*Helicobacter pylori* is a Gram-negative, microaerophilic bacterium that inhabits the gastric lining of almost half of the world’s population [1]. It is a major etiological agent of chronic gastritis [2], peptic ulcers, and gastric adenocarcinoma. *H. pylori* is classified as a Group I carcinogen by the World Health Organization [3].

*H. pylori* infection is typically treated with a combination of antibiotics, including clarithromycin, amoxicillin, or metronidazole, in conjunction with proton pump inhibitors. However, the increasing prevalence of antibiotic-resistant strains has significantly reduced treatment efficacy worldwide [1,4]. Beyond treatment difficulties, the widespread use of antibiotics to manage *H. pylori* may contribute to broader antibiotic resistance by disrupting the gut microbiota and promoting the selection of resistant strains in non-target bacterial populations [5,6]. These challenges render alternative or adjunctive therapies, such as plant-derived extracts with antimicrobial or microbiota-modulating properties, necessary.

Marine-derived natural products, particularly edible seaweeds, have attracted increasing attention due to their diverse phytochemical compositions and associated health benefits [7,8,9]. Among these, *Caulerpa lentillifera*, commonly known as sea grape or green caviar, is widely consumed in coastal regions of Southeast Asia [7,10]. In Thailand, its cultivation—particularly in southern provinces such as Krabi, Phetchaburi, and Phuket—is actively supported by the Department of Fisheries as part of a sustainable aquaculture strategy and rural economic development initiative [11,12]. With monthly yields exceeding 25 tons in some communities [13], farm-grown *C. lentillifera* represents a promising resource for the development of functional foods.

In addition to its nutritional value, *C. lentillifera* contains a wide array of bioactive compounds, including amino acids, polysaccharides, phenolic acids, terpenoids, and sulfated metabolites [7,10]. It exhibits properties consistent with prebiotic activity; *C. lentillifera* is rich in dietary fiber (~17.5%, comprising 16.6% insoluble fiber) and short-chain fatty acids (SCFAs), including acetate, propionate, and butyrate. These SCFAs support intestinal health, mitigate inflammation, and enhance metabolic and cardiovascular function [11]. Moreover, supplementation with *C. lentillifera* has been associated with favorable shifts in gut microbiota composition, including increased abundances of *Akkermansia*, *Lactobacillus*, Coriobacteriaceae, and Ruminococcaceae, while suppressing pathogenic taxa such as Clostridia and Erysipelotrichia [14]. These properties support *C. lentillifera*’s role in promoting gut homeostasis and immune–metabolic health. In previous work, we observed that sea grape extracts inhibited bacterial pathogens and exhibited immunomodulatory effects. Given that one of the key challenges in gastrointestinal health is persistent *H. pylori* colonization, we sought to investigate whether sea grape extracts could offer targeted gut health benefits through anti-*H. pylori* activity.

Recent studies have demonstrated the antimicrobial potential of sea grape extracts, including activity against *H. pylori* [15]. Polysaccharides derived from *C. lentillifera* were shown to inhibit *H. pylori* growth both in vitro and in human gastric epithelial (AGS) cells. The extract reduced bacterial adhesion to AGS cells and significantly suppressed *H. pylori*-induced interleukin-8 (IL-8) secretion, suggesting anti-inflammatory effects. These findings suggest that sea grape polysaccharides could impair bacterial adherence while attenuating IL-8 and NF-κB-mediated proinflammatory responses. Sea grape extracts comprise more than polysaccharides. A recent study investigated the chemical composition, antioxidant properties, and inhibitory effects against α-glucosidase of sea grapes collected from various sites in Thailand. The authors found that sea grape extract contained amino acids, lipids, carboxylic acids, nucleic acids, steroids, terpenoids, and vitamins [16].

Given these promising findings, characterizing the bioactive constituents responsible for anti-*H. pylori* effects is essential. Moreover, the metabolic impact of sea grape extracts on *H. pylori* remains poorly understood, and the underlying mechanisms influencing bacterial transcription and metabolism have yet to be elucidated.

In this study, we aimed to characterize the chemical composition of sea grape (*C. lentillifera*) extract and evaluate its antibacterial activity against *H. pylori*. We employed ultra-high-performance liquid chromatography coupled with tandem mass spectrometry (UHPLC-MS/MS) to profile the metabolite content of the extracts and performed in vitro assays to assess their inhibitory effects. To gain mechanistic insights, we further investigated the intracellular metabolic responses of *H. pylori* exposed to sea grape extracts using untargeted metabolomics. We hypothesized that bioactive constituents in sea grape disrupt essential metabolic pathways in *H. pylori*, contributing to its antibacterial action. This integrated approach allowed us to identify unique chemical signatures, assess dose-dependent growth inhibition, and elucidate potential metabolic targets.

## 2. Results and Discussion

### 2.1. Untargeted Metabolomic Profiling of Sea Grape Extract Using LC-MS/MS

**Figure 1:**
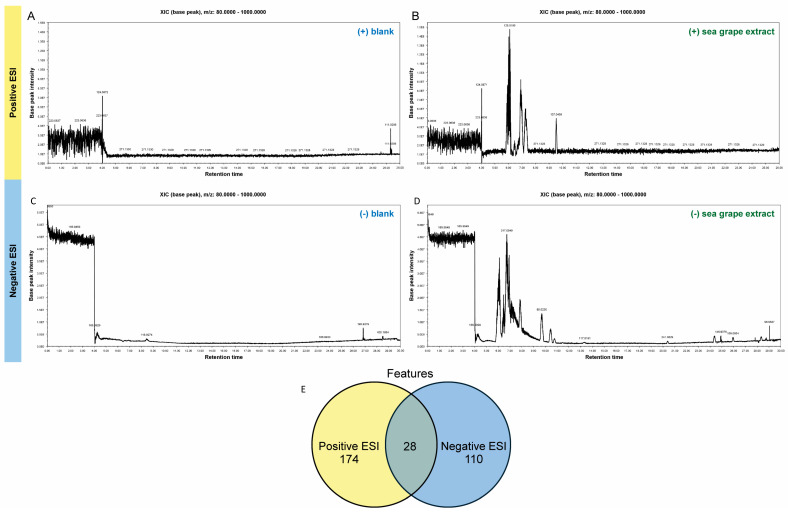
Metabolite profiling of sea grape extract using UHPLC-MS/MS. (**A**–**D**) Base peak chromatograms (BPCs) of the blank (**A**,**C**) and sea grape extract (**B**,**D**) analyzed in positive (**A**,**B**) and negative (**C**,**D**) electrospray ionization (ESI) modes. Chromatographic profiles of the extract show diverse metabolite signals compared to the blank, indicating successful extraction and detection of numerous ionizable compounds. (**E**) Venn diagram summarizing metabolite feature detection: a total of 256 features were identified, with 174 features unique to the positive ESI mode, 110 features in the negative ESI mode, and 28 features detected in both modes.

The chemical compositions of sea grape extract from farm-grown sea grape in Krabi, Thailand, were analyzed using UHPLC-MS/MS. Base peak chromatograms (BPCs) were analyzed in both positive and negative electrospray ionization (ESI) modes to assess the ionization profiles and metabolite complexity of *Caulerpa lentillifera* (sea grape) extracts compared to blank samples (Figure 1A–D). In the positive mode BPCs, the blank sample displayed a relatively flat and featureless chromatogram with low base peak intensity (~7.5 × 10^7^) and no prominent peaks beyond m/z 124.0872 and 223.0637. In contrast, the sea grape extract yielded a rich chromatographic profile, with intense and well-resolved peaks primarily eluting between 5.5 and 8.0 min, reaching intensities above 1.5 × 10^8^. Notably, peaks at m/z 128.0195, 137.0459, and 271.1328 were enriched or exclusive to the extract, indicating the presence of unique metabolites.

In negative ion mode, similar trends were observed (Figure 1C,D). The blank sample showed minimal chromatographic activity, with a nearly flat baseline interrupted by minor signals at m/z 165.9850, 116.9274, and 190.9279. Conversely, the sea grape BPC revealed several distinct peaks, with the most intense signals appearing between 6.0 and 10.0 min and peaking at m/z 317.0549. Additional ions such as m/z 89.0230, 117.0181, and 241.0829 were observed exclusively in the sea grape extract, further underscoring its chemical richness.

Using the peak identification tool, we found a total of 256 metabolite features detected across both positive (174 features) and negative (110 features) electrospray ionization (ESI) modes, with 28 metabolites detected in both ionization modes (Figure 1E; Table S1). Features commonly detected in both positive and negative ionization modes revealed a predominance of nucleotide derivatives and dipeptides, reflecting the complex biochemical composition of *C. lentillifera*. Identified purine and pyrimidine derivatives are involved in nucleic acid metabolism and may contribute to cellular signaling and antimicrobial defense [17]. Concurrently, the presence of dipeptides such as Glu-Leu, Leu-Leu, Ala-Phe, and Asp-Phe is consistent with previous reports of peptide-rich marine algae and may be linked to bioactivities including antioxidant and immunomodulatory effects [18]. Additional annotated compounds—such as riboflavin, glutamate, succinic acid, and hydroxybutyric acid—are associated with redox metabolism, the TCA cycle, and neuroactive pathways, suggesting potential roles in gut health modulation [19].

**Figure 2:**
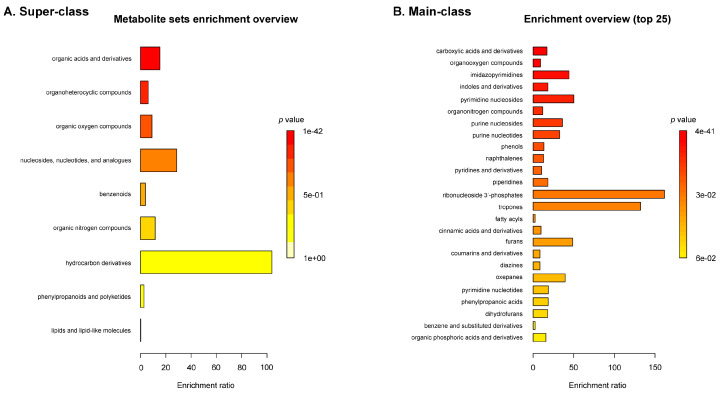
Enrichment analysis of chemical classes in sea grape extract. Detected metabolites were subjected to chemical class enrichment analysis using the MetaboAnalyst 6.0 platform. (**A**) Enrichment overview based on chemical super-classes, comprising 39 predefined metabolite sets. (**B**) Enrichment overview of the top 25 main classes out of 617 sets. Bar lengths represent the enrichment ratio, indicating the degree of overrepresentation within each class, while bar color reflects the associated *p*-value, with darker red indicating greater statistical significance.

The enrichment analysis in Figure 2 highlights the chemical diversity of metabolites in *C. lentillifera*, with dominant classes including carboxylic acids and derivatives, organo-oxygen compounds, imidazopyrimidines, indoles and derivatives, pyrimidine and purine nucleosides, organonitrogen compounds, and phenols (*p* < 0.001; FDR < 0.05). These enriched classes are consistent with previously reported GC-MS studies in *C. racemosa*, which identified several bioactive compounds such as 3,7,11,15-tetramethyl-2-hexadecen-1-ol (a diterpenoid alcohol), 3-hexadecene (an unsaturated hydrocarbon), and phthalic acid (a benzenoid dicarboxylic acid) [20]. Such compounds are known to exhibit antioxidant, antimicrobial, anticancer, and anti-mutagenic properties, supporting the potential functional relevance of the enriched chemical classes observed in our study. Notably, the presence of indole and phenol derivatives, along with nucleotide-related metabolites, suggests potential bioactivity linked to oxidative stress modulation and nucleic acid metabolism, aligning with the pharmacological profiles reported in related green algae species.

### 2.2. Sea Grape Extract Exhibits Selective Antibacterial Activity

In this study, we investigated the antibacterial activity of sea grape extract against a panel of bacterial species, including *Escherichia coli*, *Bacillus cereus*, *Salmonella* spp., *Helicobacter pylori*, and the probiotic *Lactobacillus johnsonii*. Bacterial cultures in the exponential growth phase were treated with varying concentrations of the extract for 24 h, followed by enumeration of viable cells using colony-forming unit (CFU) counts.

The results revealed species-specific responses to the sea grape extract. Statistical analysis using one-way ANOVA followed by Dunnett’s post hoc test (relative to the diluted medium control) showed that *B. cereus* and *H. pylori* were significantly inhibited by the extract (*p* < 0.05), whereas *Salmonella* spp. exhibited only minor growth reduction. Interestingly, *E. coli* displayed a slight increase in CFU counts, suggesting a potential growth-promoting or neutral effect at the tested concentrations (Figure S1). Notably, the growth of *L. johnsonii* was not adversely affected, indicating that the extract may possess selective toxicity toward pathogenic bacteria while sparing beneficial microbiota.

These findings suggest that sea grape extract exerts differential antibacterial effects, which may be attributed to the unique composition of bioactive metabolites such as sulfated polysaccharides, caulerpin, and phenolic compounds known to be present in *Caulerpa* species [20]. The pronounced inhibition of *H. pylori* is particularly significant given the clinical challenges associated with treating *H. pylori*-related infections and the rise in antibiotic resistance [21]. Natural extracts that can selectively suppress *H. pylori* while preserving beneficial microbes may represent promising candidates for alternative or adjunctive therapies. This finding prompted a closer examination of their inhibitory mechanisms against this pathogen.

### 2.3. Sea Grape Extracts Strongly Inhibit H. pylori Growth

**Figure 3:**
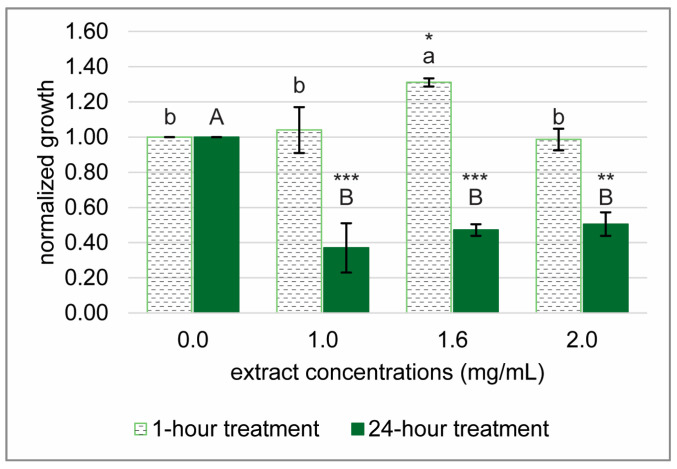
Effects of sea grape extract on *H. pylori* growth after 1 and 24 h treatments. The experiments were conducted in ½-strength BHI broth using a 12-well plate format. Bacterial growth was assessed by measuring OD_600_ and normalized to the water-treated control (0 mg/mL extract). Each experiment was performed in biological triplicates. Statistical analyses were conducted using one-way ANOVA followed by Duncan’s Multiple Range Test (DMRT) and Dunnett’s post hoc test (two-sided). Different letters above the bars indicate groups that are significantly different from each other based on DMRT (*p* < 0.05); lowercase letters correspond to the 1 h treatment group, and uppercase letters to the 24 h group. Asterisks denote significant differences between each treatment and the control according to Dunnett’s test. Significance levels: *** (*p* < 0.001), ** (*p* < 0.01), * (*p* < 0.05).

The inhibitory effect of sea grape extract on *H. pylori* growth was further confirmed using a broader range of concentrations and both short-term and long-term exposure conditions (Figure 3). A 1 h exposure to the extract did not significantly affect bacterial viability, except at 1.6 mg/mL final concentration, where a slight increase in CFU counts was observed, potentially reflecting a transient adaptive or hormetic stress response rather than enhanced growth. This finding suggests a potential transient stimulatory or hormetic response at this concentration, a phenomenon previously reported in bacterial stress response studies [22,23]. In contrast, prolonged exposure for 24 h resulted in a substantial and dose-dependent reduction in *H. pylori* colony formation, indicating potent bacteriostatic or bactericidal activity. These results support the notion that the antimicrobial efficacy of sea grape extract is time-dependent. Consequently, the 24 h treatment condition was selected for subsequent metabolomics profiling to elucidate the cellular responses and possible mechanisms of action.

To explore the biochemical basis of this antibacterial activity, we analyzed the metabolite composition of the sea grape extract. Several compounds with known or potential antimicrobial activity were detected. Notably, adenosine and nicotinic acid—both previously reported in sea grape metabolomic profiles [16]—were present. These metabolites are known to interfere with bacterial energy metabolism and redox processes [24], which may contribute to the observed growth inhibition.

Additionally, we identified several minor but potentially bioactive constituents, including hydroxyflavone, 3-sulfino-L-alanine (also known as cysteinesulfinic acid), thiophanic acid, syringic acid sulfate, and glycosylated flavonoid-like compounds. While 7-hydroxyflavone was detected at low relative abundance and is not widely reported in *Caulerpa* species, it is a flavonoid known to inhibit *H. pylori* by targeting urease activity and compromising membrane integrity [25]. Even at trace levels, such compounds may act synergistically with other phenolics and flavonoids in the extract [26,27,28].

Syringic acid, a hydroxybenzoic acid derivative, has also demonstrated antimicrobial activity, possibly through membrane disruption and induction of oxidative stress [29]. Similarly, 3-sulfino-L-alanine, a sulfur-containing amino acid derivative, may disrupt redox balance or interfere with sulfur metabolism pathways in bacteria. Although its specific activity against *H. pylori* is not well characterized, its structural similarity to cysteine analogs suggests a potential role in microbial inhibition.

### 2.4. Sea Grape Extracts Led to Metabolic Changes in H. pylori

**Figure 4:**
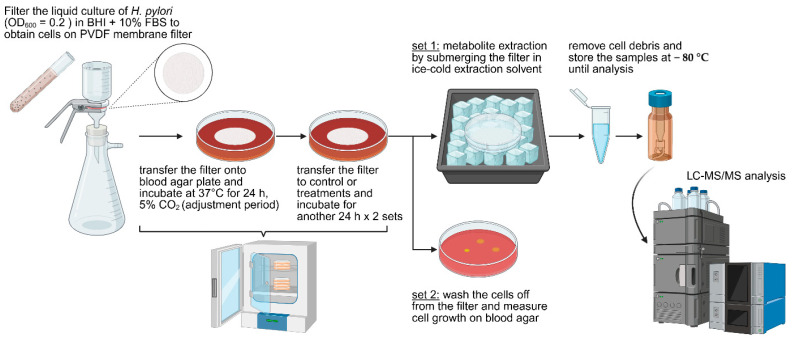
Experimental workflow for metabolomics analysis of *H. pylori* treated with sea grape extract. *H. pylori* liquid cultures were filtered through a PVDF membrane to retain bacterial cells on the surface. The membrane was first transferred onto a blood agar plate to allow cell acclimatization. After acclimation, the membrane was transferred onto treatment plates containing either sea grape extract or control medium. For each sample, two parallel setups were prepared: one for intracellular metabolite extraction and the other for bacterial growth assessment (growth results shown in Figure S2).

We employed an LC-MS/MS-based metabolomics approach to investigate how sea grape extract affects *H. pylori* at the metabolic level. To ensure rapid and efficient extraction of intracellular metabolites under defined growth conditions, we developed a filter-based culturing system. As illustrated in Figure 4, *H. pylori* cultures were first collected onto a PVDF membrane via vacuum filtration, allowing cells to remain intact on the filter surface. The membrane was placed on an agar plate. This setup allowed controlled exposure and minimized handling time. The immediate transfer of the membrane to a cold extraction solvent enabled rapid quenching of metabolic activity, preserving the metabolic state of *H. pylori* cells at the time of treatment.

**Figure 5:**
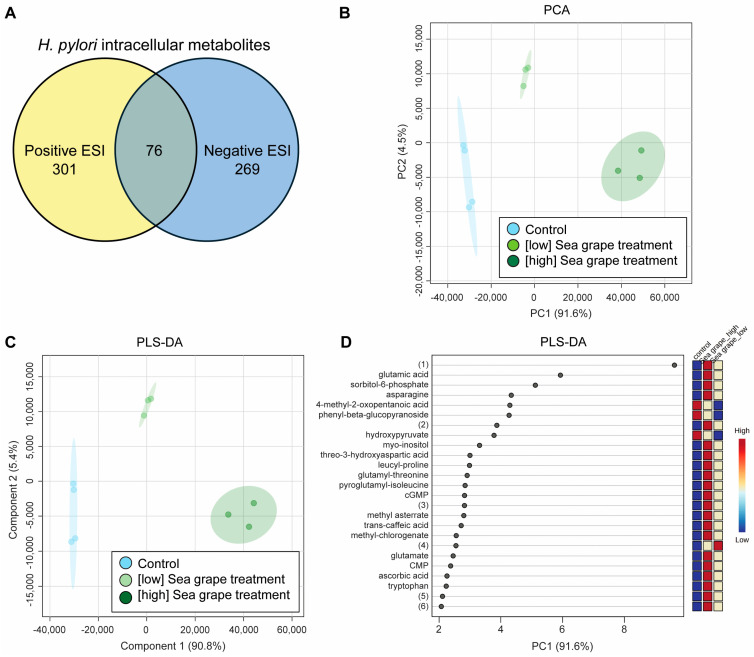
Overview of intracellular metabolite profiles of *H. pylori* treated with sea grape extract or control. (**A**) UHPLC-MS/MS analysis detected a total of 646 metabolites from *H. pylori* samples. Of these, 377 metabolites were identified in positive ESI mode and 345 in negative ESI mode, with 76 metabolites detected in both modes. (**B**) PCA 2D score plot showing the separation between sea grape-treated and control samples. (**C**) PLS-DA 2D score plot further highlighting the distinction between treatment groups. (**D**) Top 25 metabolites ranked by variable importance in projection (VIP) scores (component 1) from the PLS-DA model, indicating key metabolites contributing to group separation. Metabolite (1), 4-hydroxy-5-[(2S,3R,4S,5S,6R)-3,4,5-trihydroxy-6-(hydroxymethyl)oxan-2-yl]oxy-1H-benzo[f][2]benzofuran-3-one (C_18_H_18_O_9_); Metabolite (2), 2-formylbenzenesulfonate (contaminant); cGMP, guanosine cyclic monophosphate; Metabolite (3), 2-[5-[2-[2-[5-[2-[2-[5-[2-[2-[5-(2-hydroxybutyl)oxolan-2-yl]propanoyloxy]propyl]oxolan-2-yl]propanoyloxy]propyl]oxolan-2-yl]propanoyloxy]propyl]oxolan-2-yl]propanoic acid (C_41_H_68_O_13_); Metabolite (4), [(2S,3R,4S,5S,6R)-3,4,5-trihydroxy-6-[[(E)-3-phenylprop-2-enoyl]oxymethyl]oxan-2-yl] 3,4,5-trihydroxybenzoate (C_22_H_22_O_11_); CMP, cytidine monophosphate; Metabolite (5), EDTA (contaminant); Metabolite (6), 3-[(3S,5S,8R,10S,13R,14S,17R)-3-[4,5-dihydroxy-6-(hydroxymethyl)-3-[3,4,5-trihydroxy-6-(hydroxymethyl)oxan-2-yl]oxyoxan-2-yl]oxy-14-hydroxy-10,13-dimethyl-1,2,3,4,5,6,7,8,9,11,12,15,16,17-tetradecahydrocyclopenta[a]phenanthren-17-yl]-2H-furan-5-one (C_35_H_54_O_14_).

LC-MS/MS analysis followed by peak identification revealed 646 metabolites across both ionization modes, with 377 metabolites detected in positive ESI and 345 in negative ESI. Among these, 76 metabolites were identified in both modes (Figure 5A). Statistical analysis using ANOVA with Fisher’s LSD post hoc test (FDR < 0.05) indicated that 612 metabolites were present at significantly different levels among *H. pylori* treated with low- and high-dose sea grape extracts compared to control.

Both unsupervised PCA and supervised PLS-DA revealed clear clustering of intracellular metabolite profiles according to treatment groups, supporting the presence of treatment-specific metabolic signatures (Figure 5B,C). The top 25 metabolites contributing most to group separation, as determined by variable importance in projection (VIP) scores from PLS-DA component 1, are shown in Figure 5D.

Enrichment analysis of the top-ranked metabolites contributing to components 1 and 2 revealed significant overrepresentation of several metabolic pathways (Figure S3). Notably, the most enriched pathways included alanine, aspartate, and glutamate metabolism; glyoxylate and dicarboxylate metabolism; pyrimidine metabolism; nitrogen metabolism; and purine metabolism (*p* < 0.001; FDR < 0.05). Among these, nitrogen metabolism exhibited the highest enrichment ratio (30) (Figure S3), indicating a substantial involvement of nitrogen-related intermediates in the metabolic response. The enrichment of amino acid biosynthesis and degradation pathways—such as those involving arginine, valine, leucine, and isoleucine—further underscores the modulation of primary nitrogen and energy metabolism [30]. Additional pathways such as the citrate cycle (TCA cycle), glutathione metabolism, and purine/pyrimidine turnover also appeared among the top hits, suggesting coordinated alterations in both redox balance and nucleotide biosynthesis [31,32]. These findings echoed a broad metabolic reprogramming associated with *H. pylori* cellular responses to exposure to sea grape extract.

**Figure 6:**
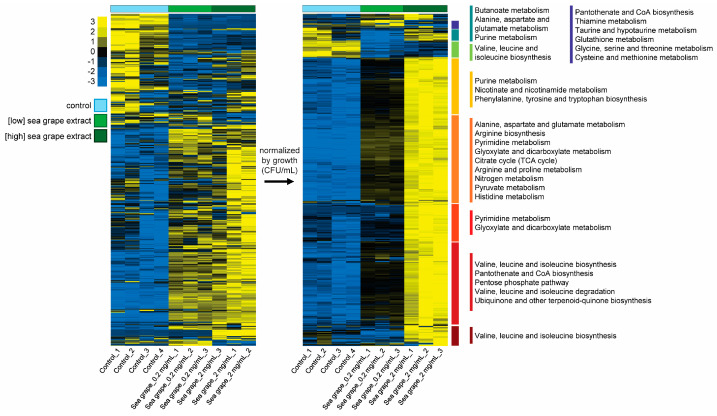
Heatmap of intracellular metabolite profiles in *H. pylori* treated with sea grape extracts. Intracellular metabolite intensities from *H. pylori* treated with control, low-dose, or high-dose sea grape extract were standardized using z-score normalization ($\frac{x-\mu }{\sigma }$). Complete-linkage hierarchical clustering was performed to group samples and metabolites based on similarity. Yellow indicates relatively high metabolite abundance, whereas blue indicates low abundance. To account for the growth-inhibitory effects of sea grape extract, metabolite intensities were further normalized to the relative bacterial growth, and the resulting growth-adjusted heatmap is shown on the right. Metabolite pathway enrichment analysis was performed using KEGG pathway sets in MetaboAnalyst 6.0.

To further visualize treatment effects, we constructed heatmaps with complete-linkage hierarchical clustering. After normalizing metabolite abundance by the relative growth of *H. pylori*, we observed that most intracellular metabolites appeared elevated in high-dose sea grape extract-treated samples compared to control (Figure 6), indicating a dose-dependent metabolic perturbation.

The metabolomic profile of *H. pylori* under sea grape extract treatment suggests a complex response combining central metabolic stress, redox imbalance, and altered nitrogen metabolism. Overall, the accumulation of glycolytic and pentose phosphate pathway (PPP) intermediates implies compensatory metabolic rewiring. The selective depletion of succinate and nitrogenous compounds reflects targeted inhibition of energy and nitrogen handling pathways. These patterns are consistent with the known bioactivities of phytochemicals, including phenolics, terpenoids, and polysaccharides, which were sea grape-derived. These chemical constituents can disrupt bacterial redox homeostasis, enzymatic functions, and membrane integrity [33,34].

Central metabolic rewiring—Despite biomass-normalized data showing a general increase in central carbon metabolites following *Helicobacter pylori* exposure to sea grape (*Caulerpa lentillifera*) extract, notable exceptions suggest targeted metabolic disruptions. Intermediates of glycolysis, the pentose phosphate pathway (PPP), and the tricarboxylic acid (TCA) cycle—such as glucose-6-phosphate, citrate, and malate—were elevated, consistent with stress-induced bottlenecks or compensatory flux activation. However, succinate, a key TCA cycle metabolite, was significantly depleted across both treatment concentrations, indicating a possible interruption in the oxidative segment of the TCA cycle or a redirection of succinate into alternative pathways.

A plausible explanation was that succinate dehydrogenase (SDH), a redox-sensitive enzyme and part of both the TCA cycle and electron transport chain, might be inhibited by oxidative stress or phenolic interference, limiting succinate accumulation [35]. Alternatively, increased consumption of succinate for reactive oxygen species (ROS) scavenging or as an electron donor under redox pressure could explain its depletion. The selective decrease of succinate against a background of elevated TCA intermediates suggests that sea grape bioactives may impair electron transport-linked respiration, consistent with previously reported effects of marine polyphenols on bacterial energetics [36].

Nitrogen metabolism—In this analysis, we observed changes in the levels of all amino acids except glycine, which was undetectable due to its molecular mass falling below the scan range (m/z < 80). Additionally, we identified several modified amino acids, including acetylated and methylated derivatives, as well as small peptides composed of fewer than five amino acids (Table S2). Most of these compounds exhibited dose-dependent increases in relative abundance following sea grape treatment. However, many were also detected in the sea grape and mulberry extracts themselves. To avoid misinterpretation caused by extracellular carryover from the extracts, we examined the overlap between the extract composition and the intracellular metabolite profiles. A total of 115 overlapping metabolites were identified, which were excluded from further interpretation as *H. pylori*-derived metabolites.

Following the removal of these overlapping metabolites, we reanalyzed the data and identified several metabolites that increased in a dose-dependent manner upon sea grape exposure. These were significantly enriched in pathways related to alanine, aspartate, and glutamate metabolism; arginine biosynthesis; pyrimidine metabolism; and the TCA cycle (highlighted in orange and red in the heat map, Figure 6; *p* < 0.01, FDR < 0.05).

Furthermore, we observed enrichment in one-carbon metabolism pathways. Key metabolites contributing to this enrichment included N-formylglycine, maleic acid, malonic acid, Ala-Ala, citrate, D-erythrose-4-phosphate, asparagine, glutamine, folic acid, arginine, fumarate, alanine, aspartic acid, ornithine, and various nucleotide derivatives. Their modulation suggests a metabolic adaptation to the bioactive stress imposed by the extract. Importantly, aspartate and glutamate serve as direct anaplerotic inputs into the TCA cycle—via transamination reactions forming oxaloacetate and α-ketoglutarate, respectively [37]. Consistent with this, we observed elevated levels of several TCA cycle intermediates in treated cells, except for succinate. These changes may represent a compensatory response to maintain redox homeostasis and energy production under phytochemical-induced stress.

One-carbon metabolism fuels both purine and pyrimidine biosynthesis [38]. Interestingly, our data revealed specific enrichment in pyrimidine biosynthesis pathways, with no corresponding increase in purine metabolism. This selective enrichment may reflect a shift in cellular priorities, where pyrimidine synthesis is critical for DNA repair and replication under stress conditions [39] induced by the extracts. In contrast, de novo purine biosynthesis is more energetically costly and requires multiple one-carbon transfers (e.g., via 10-formyl-THF), making it less favorable when energy and redox balance are compromised. This imbalance could also suggest cell cycle arrest or a checkpoint response, where the bacterium prioritizes maintaining DNA integrity (thymidine supply) over initiating new rounds of replication. Such metabolic reprogramming aligns with known bacterial stress responses [40] and highlights the potential of *Caulerpa* extracts to disrupt nucleotide homeostasis and exert antimicrobial pressure through metabolic interference.

## 3. Materials and Methods

### 3.1. Biomass and Extract Preparation

Fresh *Caulerpa lentillifera* (commonly known as sea grape or green caviar) was sourced from a commercial aquaculture farm, Baan Sarai Khonnok Company Limited, in Krabi Province, Thailand. Upon arrival, the seaweed was thoroughly rinsed with distilled water to remove external contaminants and debris. The cleaned biomass was then dried using a stepwise, temperature-controlled hot air-drying process designed to preserve the material’s physical and chemical integrity while reducing the moisture content to below 10%. After drying, the sea grape was ground into a fine powder using a mechanical grinder. Aqueous extraction was carried out using a proprietary protocol optimized to retain bioactive constituents; full methodological details are withheld due to a pending petty patent application. The resulting extract was clarified by sequential filtration and centrifugation to remove insoluble particulates. The final supernatant was adjusted to 30 mg/mL with LC-MS-grade water and stored at −20 °C until use in chemical composition analyses and antibacterial assays. For bioactivity testing, sea grape extracts were further diluted to final concentrations of 0.2 and 2 mg/mL. All chemicals used in this study were of LC grade and purchased from Sigma-Aldrich (Singapore), unless otherwise stated.

### 3.2. Strains and Media

*Helicobacter pylori* ATCC 43504™ was maintained in brain heart infusion (BHI) broth (Oxoid, Basingstoke, UK) supplemented with 10% heat-inactivated fetal bovine serum (FBS; Gibco™, Invitrogen, Waltham, MA, USA) under microaerophilic conditions. The bacterial strain was incubated at 37 °C in a CO_2_ chamber to maintain optimal atmospheric conditions. Alternatively, for anaerobic cultivation, an anaerobic jar equipped with BD GasPak™ system (BD, Singapore) was utilized.

BHI broth was used for maintaining *H. pylori* and for assaying the inhibitory effects of sea grape and mulberry extracts against the bacterium. Meanwhile, Columbia agar with 5% Sheep blood (BD BBL™, Singapore) was used for the metabolomics experiment where the effects of sea grape and mulberry extracts on *H. pylori*’s intracellular metabolites were examined. The latter experimental setup is described below in more detail.

### 3.3. Antibacterial Property Evaluation

The antibacterial activities of sea grape and mulberry extracts were initially evaluated against a panel of bacterial strains, including *Escherichia coli* DE3, *Bacillus cereus* ATCC 14579™, *Salmonella* sp., *H. pylori*, and *Lactobacillus johnsonii* CK-3, using a ½-strength LB broth in a 12-well plate format. The bacterial cultures were grown to an optical density at 600 nm (OD_600_) of 0.250–0.400 and then inoculated into each well at a 1:1000 dilution. Each well contained 2 mL of ½ LB broth supplemented with either 0.2 mg/mL or 2 mg/mL of sea grape or mulberry extract. The cultures were incubated overnight at 37 °C prior to growth assessment.

For the evaluation of inhibitory activity against *H. pylori*, bacterial cultures were prepared in brain heart infusion (BHI) broth and dispensed into sterile 12-well plates at a final volume of 4 mL per well. Sea grape and mulberry extracts were tested at final concentrations of 0.2 mg/mL and 2 mg/mL, respectively. Sterile distilled water was used as the negative control, and all treatments were performed in triplicate. The inoculated plates were incubated at 37 °C for 24–48 h under CO_2_-enriched conditions. Bacterial growth inhibition was assessed at both 24 h and 48 h time points by measuring OD_600_ and performing the drop plate method for colony enumeration.

### 3.4. Chemical Composition Analyses of Sea Grape Extract

The samples were stored at −20 °C until analyses and were filtered through 0.22 µm nylon syringe filters to eliminate small particles/debris. The resultant samples were subject to LC-MS/MS analysis. In this study, the UHPLC-MS system consisted of the Dionex Ultimate 3000 UHPLC system coupled with the Orbitrap Q Exactive Focus mass spectrometer (Thermo Scientific, Waltham, MA, USA). Ten μL of each sample was injected with an autosampler system kept at 4 °C. The liquid separation was achieved using a C18 column (Poroshell 120 EC-C18, 2.7 μm × 100 mm, Agilent, Santa Clara, CA, USA). Gradient chromatography was achieved using 0.1% formic acid as Solvent A and acetonitrile with 0.1% formic acid as Solvent B. The gradient was as follows: 5% B from 0.0–5.0 min, 5–95% B from 5.0–25.0 min, 95–100% B from 25.0–26.0 min, 100% B from 26.0–35.0 min, 100–5% B from 35.0–36.0 min, and 5% B until 45.0 min. The flow rate was 0.25 mL/min. The total run time was 45 min. Samples were run using positive electrospray ionization (ESI). Data acquisition was achieved in a full scan/ddMS2 mode. The full scan resolution was 35,000 with an 85–1000 m/z scan range. The discovery ddMS2 was set at a 17,500 resolution with a 3.0 isolation window; the collision energy (NCE) was 15. The HESI-II working parameters were as follows: spray voltage at 3 kV, capillary temperature at 350 °C, sheath gas flow rate at 30 psi, auxiliary gas flow rate at 10 au, and auxiliary gas heater temperature at 300 °C. LC-grade water was used as blank, and amino acid standard mix (AAS18, Sigma-Aldrich, St. Louis, MO, USA) was used as external standards.

Experimental MS data were exported in the mzXML format and used for metabolite identification. Base peak chromatograms (BPCs) were generated using MZMine2 to visualize and compare the overall metabolite profiles between the blank and sea grape extract samples. Metabolite peaks were annotated using MassBank of North America (MoNA) libraries [41,42] and validated using MAVEN2 (metabolomics analysis and visualization engine) [43] with a mass error of ±10 ppm. The metabolites identified with MAVEN2 were validated manually based on peak integrity. The validated data were used for statistical analysis performed on the MetaboAnalyst 6.0 platform “https://www.metaboanalyst.ca/MetaboAnalyst/ (accessed on 22 April 2025).”

### 3.5. Examination of Metabolic Response of H. pylori Treated with Sea Grape Extract

To evaluate the effects of sea grape extract on *Helicobacter pylori*, the bacterium was cultured in BHI broth supplemented with 10% FBS. The sample collection protocol was adapted from Bennett et al. [44] and Pisithkul et al. [45], with modifications. Briefly, cultures in the exponential growth phase (OD_600_ 0.2–0.3; approximately 2–4 × 10^6^ CFU/mL) were harvested by vacuum filtration through a 0.22 µm polyvinylidene fluoride (PVDF) membrane (Merck, Singapore). The filter membrane containing the bacterial cells was immediately placed, cell-side up, onto Columbia agar supplemented with 5% sheep blood. This setup allowed the cells to continue growing on the membrane surface, accessing nutrients diffusing from the agar medium.

The plates were incubated at 37 °C in a humidified, 5% CO_2_ incubator for 24 h to allow the cells to acclimate to the membrane-supported growth conditions. After this pre-incubation, the membrane was transferred to a fresh Columbia blood agar plate, either containing sea grape extract at final concentrations of 0.2 or 2 mg/mL, or to a control plate without extract. The transfer of the membrane was performed rapidly (within a few seconds) to minimize environmental fluctuations. Plates were then incubated for an additional 24 h under the same conditions.

For metabolite extraction, the PVDF membrane was swiftly transferred from the agar plate into a pre-chilled solvent mixture of methanol:acetonitrile:water (40:40:20, *v*/*v*/*v*) prepared in a small Petri dish placed on dry ice. The total volume of the extraction solvent was 1.5 mL. The extractant was then transferred to a 1.5 mL tube and centrifuged to remove cell debris. The resulting supernatant, containing the intracellular metabolites of *H. pylori*, was stored at −80 °C until subsequent LC-MS/MS analysis.

## 4. Conclusions

This study demonstrated that *Caulerpa lentillifera* (sea grape) extract exerts potent, dose- and time-dependent inhibitory effects on *Helicobacter pylori*, as evidenced by cell counts and metabolomic profiling. Using UHPLC-MS/MS, we identified multiple classes of potentially bioactive compounds, including flavonoids, phenolic acids, and sulfur-containing amino acid derivatives. Notably, adenosine, nicotinic acid, syringic acid sulfate, hydroxyflavone, and 3-sulfino-L-alanine were detected—compounds known or predicted to disrupt bacterial metabolism, membrane function, and redox balance. These metabolites likely contribute to the observed metabolic alterations in *H. pylori*, including reduced succinate levels and elevations in glutamate and leucine, implicating interference with the TCA cycle and amino acid biosynthesis. Taken together, our findings highlight *C. lentillifera* as a promising source of antimicrobial compounds with selective activity against *H. pylori*. However, further development is necessary to standardize bioactive composition and enhance stability and bioavailability of the extract. Future studies should also validate the efficacy and safety of sea grape-derived compounds in in vivo models of gastric infection, and ultimately, in human clinical trials. Such efforts will be essential for translating this marine-derived phytochemical resource into a viable therapeutic or nutraceutical intervention against *H. pylori*-associated diseases.

## Data Availability

All data supporting the findings of this study are available within the article and its Supplementary Materials.

## Supplementary Materials

The following supporting information can be downloaded at https://www.mdpi.com/article/10.3390/md23070282/s1. Table S1: Annotated metabolite profiles of sea grape extract based on UHPLC-MS/MS analysis; Table S2: Differential intracellular metabolite profiles of *H. pylori* following sea grape extract treatment; Figure S1: Antibacterial activity of sea grape extract against selected bacterial species; Figure S2: *H. pylori* growth inhibition by sea grape extracts in the metabolomics experiment setup; Figure S3: Pathway enrichment analysis of top-ranked metabolites in sea grape extract-treated *H. pylori*.

## References

[1] Chen Y.-C., Malfertheiner P., Yu H.-T., Kuo C.-L., Chang Y.-Y., Meng F.-T., Wu Y.-X., Hsiao J.-L., Chen M.-J., Lin K.-P. (2024). Global prevalence of *Helicobacter pylori* infection and incidence of gastric cancer between 1980 and 2022. Gastroenterology.

[2] Mahboobi R., Fallah F., Yadegar A., Dara N., Aghdam M.K., Asgari B., Hakemi-Vala M. (2022). Expression analysis of miRNA-155 level in *Helicobacter pylori* related inflammation and chronic gastritis. Iran. J. Microbiol..

[3] Malfertheiner P., Mégraud F., O’Morain C.A., Gisbert J.P., Kuipers E.J., Axon A.T., Bazzoli F., Gasbarrini A., Atherton J., Graham D.Y. (2017). Management of *Helicobacter pylori* infection—The Maastricht V/Florence consensus report. Gut.

[4] Essaidi I., Bounder G., Jouimyi R.M., Boura H., Elyounsi I., Kheir F.-Z., Benomar H., Badre W., Zerouali K., Maachi F. (2022). Comparative study of *Helicobacter pylori* resistance to clarithromycin and metronidazole and its association with epidemiological factors in a moroccan population. Asian Pac. J. Cancer Prev..

[5] Zhao M., Zhang Y., Liu S., Wang F., Zhang P. (2025). Eradication of *Helicobacter pylori* reshapes gut microbiota and facilitates the evolution of antimicrobial resistance through gene transfer and genomic mutations in the gut. BMC Microbiol..

[6] Olesen S.W., Barnett M.L., MacFadden D.R., Brownstein J.S., Hernández-Díaz S., Lipsitch M., Grad Y.H. (2018). The distribution of antibiotic use and its association with antibiotic resistance. eLife.

[7] Syakilla N., George R., Chye F.Y., Pindi W., Mantihal S., Wahab N.A., Fadzwi F.M., Gu P.H., Matanjun P. (2022). A review on nutrients, phytochemicals, and health benefits of green seaweed, *Caulerpa lentillifera*. Foods.

[8] Wang H.-N., Sun S.-S., Liu M.-Z., Yan M.-C., Liu Y.-F., Zhu Z., Zhang Z. (2022). Natural bioactive compounds from marine fungi (2017–2020). J. Asian Nat. Prod. Res..

[9] Mandal A.K., Parida S., Behera A.K., Adhikary S.P., Lukatkin A.A., Lukatkin A.S., Jena M. (2025). Seaweed in the diet as a source of bioactive metabolites and a potential natural immunity booster: A comprehensive review. Pharmaceuticals.

[10] Chen X., Sun Y., Liu H., Liu S., Qin Y., Li P. (2019). Advances in cultivation, wastewater treatment application, bioactive components of *Caulerpa lentillifera* and their biotechnological applications. PeerJ.

[11] du Preez R., Majzoub M.E., Thomas T., Panchal S.K., Brown L. (2020). *Caulerpa lentillifera* (sea grapes) improves cardiovascular and metabolic health of rats with diet-induced metabolic syndrome. Metabolites.

[12] Amornlerdpison D., Yoojam S., Mengumphan K., Lailerd N. (2023). Functional ingredients on creating value added in sea grape, *Caulerpa lentillifera*. J. Agric. Res. Ext..

[13] Srinorasing T., Chirasuwan N., Bunnag B., Chaiklahan R. (2021). Lipid extracts from *Caulerpa lentillifera* waste: An alternative product in a circular economy. Sustainability.

[14] Sun Y., Liu Y., Ai C., Song S., Chen X. (2019). *Caulerpa lentillifera* polysaccharides enhance the immunostimulatory activity in immunosuppressed mice in correlation with modulating gut microbiota. Food Funct..

[15] Le B., Do D.T., Nguyen H.M., Do B.H., Le H.T. (2022). Preparation, characterization, and anti-adhesive activity of Sulfate polysaccharide from *Caulerpa lentillifera* against *Helicobacter pylori*. Polymers.

[16] Koodkaew I., Pitakwongsaporn S., Jarussophon N., Wichachucherd B. (2024). Chemical Profile, Antioxidant activity and α-glucosidase inhibition of sea grape *Caulerpa lentillifera* collected from different sites in Thailand. Trends Sci..

[17] Rodríguez-Rojas A., Nath A., El Shazely B., Santi G., Kim J.J., Weise C., Kuropka B., Rolff J. (2020). Antimicrobial peptide induced-stress renders *Staphylococcus aureus* susceptible to toxic nucleoside analogs. Front. Immunol..

[18] Samarakoon K., Jeon Y.-J. (2012). Bio-functionalities of proteins derived from marine algae—A review. Food Res. Int..

[19] Dalile B., Van Oudenhove L., Vervliet B., Verbeke K. (2019). The role of short-chain fatty acids in microbiota–gut–brain communication. Nat. Rev. Gastroenterol. Hepatol..

[20] Palaniyappan S., Sridhar A., Kari Z.A., Téllez-Isaías G., Ramasamy T. (2023). Evaluation of phytochemical screening, pigment content, *in vitro* antioxidant, antibacterial potential and GC-MS metabolite profiling of green seaweed *Caulerpa racemosa*. Mar. Drugs.

[21] Tshibangu-Kabamba E., Yamaoka Y. (2021). *Helicobacter pylori* infection and antibiotic resistance—From biology to clinical implications. Nat. Rev. Gastroenterol. Hepatol..

[22] Calabrese E.J., Agathokleous E., Kapoor R., Kozumbo W.J., Rattan S.I. (2019). Re-analysis of herbal extracts data reveals that inflammatory processes are mediated by hormetic mechanisms. Chem.-Biol. Interact..

[23] Ohno H., Miyoshi S., Araho D., Kanamoto T., Terakubo S., Nakashima H., Tsuda T., Sunaga K., Amano S., Ohkoshi E. (2014). Efficient utilization of licorice root by alkaline extraction. In Vivo.

[24] Huang J., Cui L., Natarajan M., Barone P.W., Wolfrum J.M., Lee Y.H., Rice S.A., Springs S.L. (2022). The ratio of nicotinic acid to nicotinamide as a microbial biomarker for assessing cell therapy product sterility. Mol. Ther. Methods Clin. Dev..

[25] Bonifácio B.V., dos Santos Ramos M.A., Da Silva P.B., Bauab T.M. (2014). Antimicrobial activity of natural products against *Helicobacter pylori*: A review. Ann. Clin. Microbiol. Antimicrob..

[26] Tan A., Scortecci K.C., Boylan F. (2025). A review on flavonoids as anti-*Helicobacter pylori* agents. Appl. Sci..

[27] Ivyna de Araújo Rêgo R., Guedes Silvestre G.F., Ferreira de Melo D., Albino S.L., Pimentel M.M., Silva Costa Cruz S.B., Silva Wurzba S.D., Rodrigues W.F., Goulart de Lima Damasceno B.P., Cançado Castellano L.R. (2022). Flavonoids-rich plant extracts against *Helicobacter pylori* infection as prevention to gastric cancer. Front. Pharmacol..

[28] Sharaf M., Arif M., Hamouda H.I., Khan S., Abdalla M., Shabana S., Rozan H.E., Khan T.U., Chi Z., Liu C. (2022). Preparation, urease inhibition mechanisms, and anti-*Helicobacter pylori* activities of hesperetin-7-rhamnoglucoside. Curr. Res. Microb. Sci..

[29] Shi C., Sun Y., Zheng Z., Zhang X., Song K., Jia Z., Chen Y., Yang M., Liu X., Dong R. (2016). Antimicrobial activity of syringic acid against *Cronobacter sakazakii* and its effect on cell membrane. Food Chem..

[30] Miyakoshi M. (2024). Multilayered regulation of amino acid metabolism in *Escherichia coli*. Curr. Opin. Microbiol..

[31] Xiang J., Tian S.-q., Wang S.-w., Liu Y.-l., Li H., Peng B. (2024). Pyruvate abundance confounds aminoglycoside killing of multidrug-resistant bacteria via glutathione metabolism. Research.

[32] Shibayama K., Wachino J.i., Arakawa Y., Saidijam M., Rutherford N.G., Henderson P.J. (2007). Metabolism of glutamine and glutathione via γ-glutamyltranspeptidase and glutamate transport in *Helicobacter pylori*: Possible significance in the pathophysiology of the organism. Mol. Microbiol..

[33] Christopher A., Sarkar D., Shetty K. (2021). Elicitation of stress-induced phenolic metabolites for antimicrobial applications against foodborne human bacterial pathogens. Antibiotics.

[34] Makarewicz M., Drożdż I., Tarko T., Duda-Chodak A. (2021). The interactions between polyphenols and microorganisms, especially gut microbiota. Antioxidants.

[35] Silva de Abreu T., Braga M.A., Trento M.V.C., Konig I.F.M., Machado G.H.A., Ferreira da Cunha E.F., Marcussi S. (2021). Gallic and vanillic acids as promising succinate dehydrogenase inhibitors and antigenotoxic agents. Rev. Bras. Farmacogn..

[36] Obrenovich M., Li Y., Tayahi M., Reddy V.P. (2022). Polyphenols and small phenolic acids as cellular metabolic regulators. Curr. Issues Mol. Biol..

[37] Chandel N.S. (2021). Amino Acid Metabolism. Cold Spring Harb. Perspect. Biol..

[38] Shuvalov O., Petukhov A., Daks A., Fedorova O., Vasileva E., Barlev N.A. (2017). One-carbon metabolism and nucleotide biosynthesis as attractive targets for anticancer therapy. Oncotarget.

[39] Newman A.C., Maddocks O.D.K. (2017). One-carbon metabolism in cancer. Br. J. Cancer.

[40] Xu J., Zhao N., Meng X., Li J., Zhang T., Xu R., Wei X., Fan M. (2023). Transcriptomic and metabolomic profiling uncovers response mechanisms of *Alicyclobacillus acidoterrestris* DSM 3922T to acid stress. Microbiol. Spectr..

[41] Horai H., Arita M., Kanaya S., Nihei Y., Ikeda T., Suwa K., Ojima Y., Tanaka K., Tanaka S., Aoshima K. (2010). MassBank: A public repository for sharing mass spectral data for life sciences. J. Mass Spectrom..

[42] Vaniya A., Mehta S., Wohlgemuth G., Fiehn O. MassBank of North America: Using untargeted metabolomics and multistage fragmentation mass spectral libraries to annotate natural products in plants. Proceedings of the 2nd International Plant Spectroscopy Conference.

[43] Seitzer P., Bennett B., Melamud E. (2022). MAVEN2: An updated open-source mass spectrometry exploration platform. Metabolites.

[44] Bennett B.D., Yuan J., Kimball E.H., Rabinowitz J.D. (2008). Absolute quantitation of intracellular metabolite concentrations by an isotope ratio-based approach. Nat. Protoc..

[45] Pisithkul T., Schroeder J.W., Trujillo E.A., Yeesin P., Stevenson D.M., Chaiamarit T., Coon J.J., Wang J.D., Amador-Noguez D. (2019). Metabolic remodeling during biofilm development of *Bacillus subtilis*. mBio.

