# Supplementary material for: Metabolomic Profiling and Anti-Helicobacter pylori Activity of Caulerpa lentillifera (Sea Grape) Extract
Source: Mar Drugs. 2025 Jul 7;23(7):282. doi: 10.3390/md23070282 (PMC12298704; doi:10.3390/md23070282)
Supplement: Supplementary file 1 [file marinedrugs-23-00282-s001.zip › Supplementary figures.pdf]

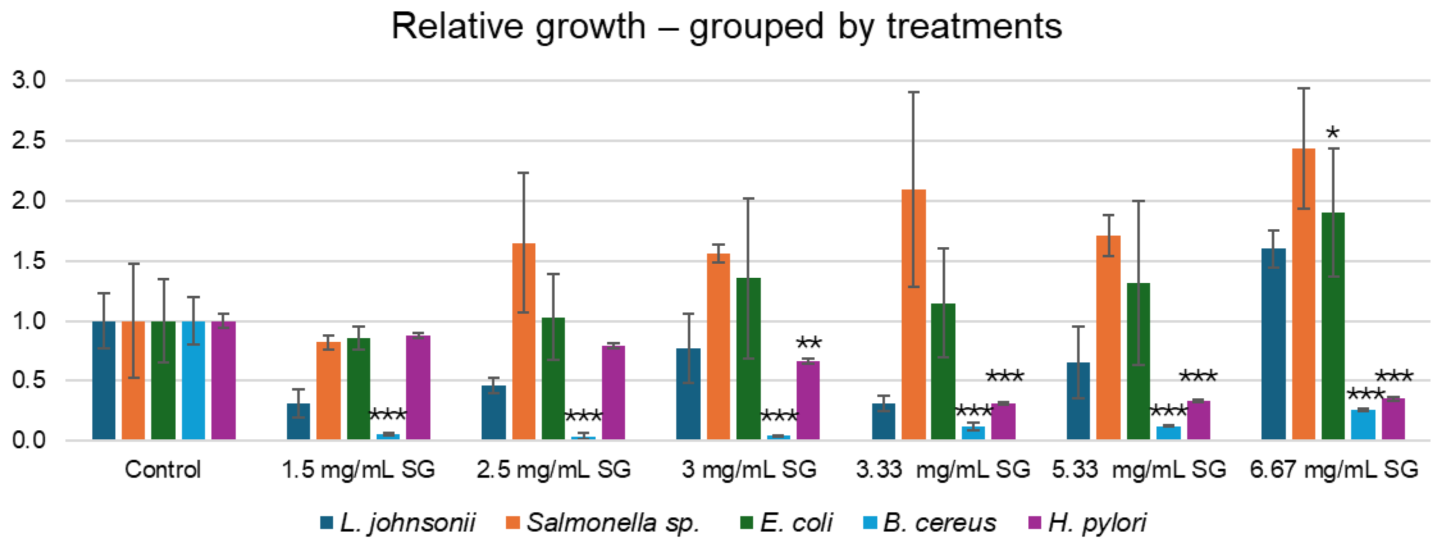

Figure S1: Antibacterial activity of sea grape extract against selected bacterial species. Five bacterial strains (*Salmonella enterica*, *Escherichia coli*, *Bacillus cereus*, *Helicobacter pylori*, and *Lactobacillus johnsonii*) were cultured in liquid media supplemented with either sea grape extract or a vehicle control in a 12-well plate format. Control cultures received 5% (v/v) of the corresponding broth medium (nutrient broth for *Salmonella*, *E. coli*, and *B. cereus*; BHI for *H. pylori*; and MRS broth for *L. johnsonii*). Treatment groups received sea grape extract at final concentrations of 1.5, 2.5, 3.0, 3.33, 5.33, and 6.67 mg/mL. Following incubation, cultures were serially diluted and plated to determine viable cell counts (CFU/mL). Bar graphs display the relative bacterial growth compared to the corresponding control. Data represent mean ± standard deviation from three biological replicates, each with technical duplicates. Significance levels: *** ($p < 0.001$), ** ($p < 0.01$), * ($p < 0.05$).

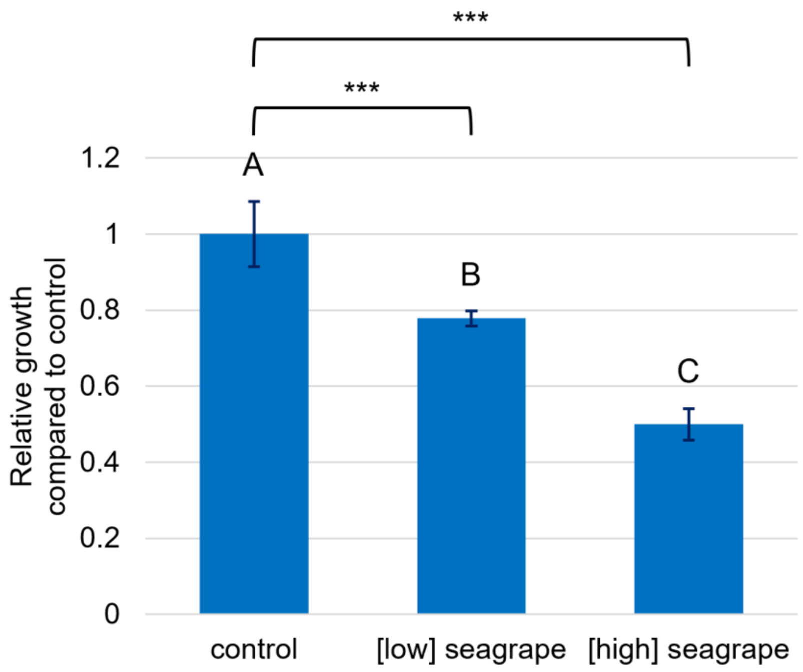

Figure S2: *H. pylori* growth inhibition by sea grape extracts in the metabolomics experiment setup. *H. pylori* cultures were treated with sea grape (*Caulerpa lentillifera*) extracts at two concentrations (low and high), followed by washing and recovery from membrane filters. Cell viability was assessed by serial dilution and drop-plating to determine CFU/mL. Growth values were normalized to the untreated control. Data represent the mean ± standard deviation of five biological replicates performed in parallel with samples used for intracellular metabolite extraction and LC-MS/MS analysis. A dose-dependent decrease in bacterial growth was observed upon sea grape treatment. Different letters (A–C) above bars indicate groups that are significantly different from each other based on Duncan’s Multiple Range Test (DMRT, $p < 0.05$). Asterisks (***) denote statistically significant differences compared to the control according to Dunnett’s post hoc test ($p < 0.001$).

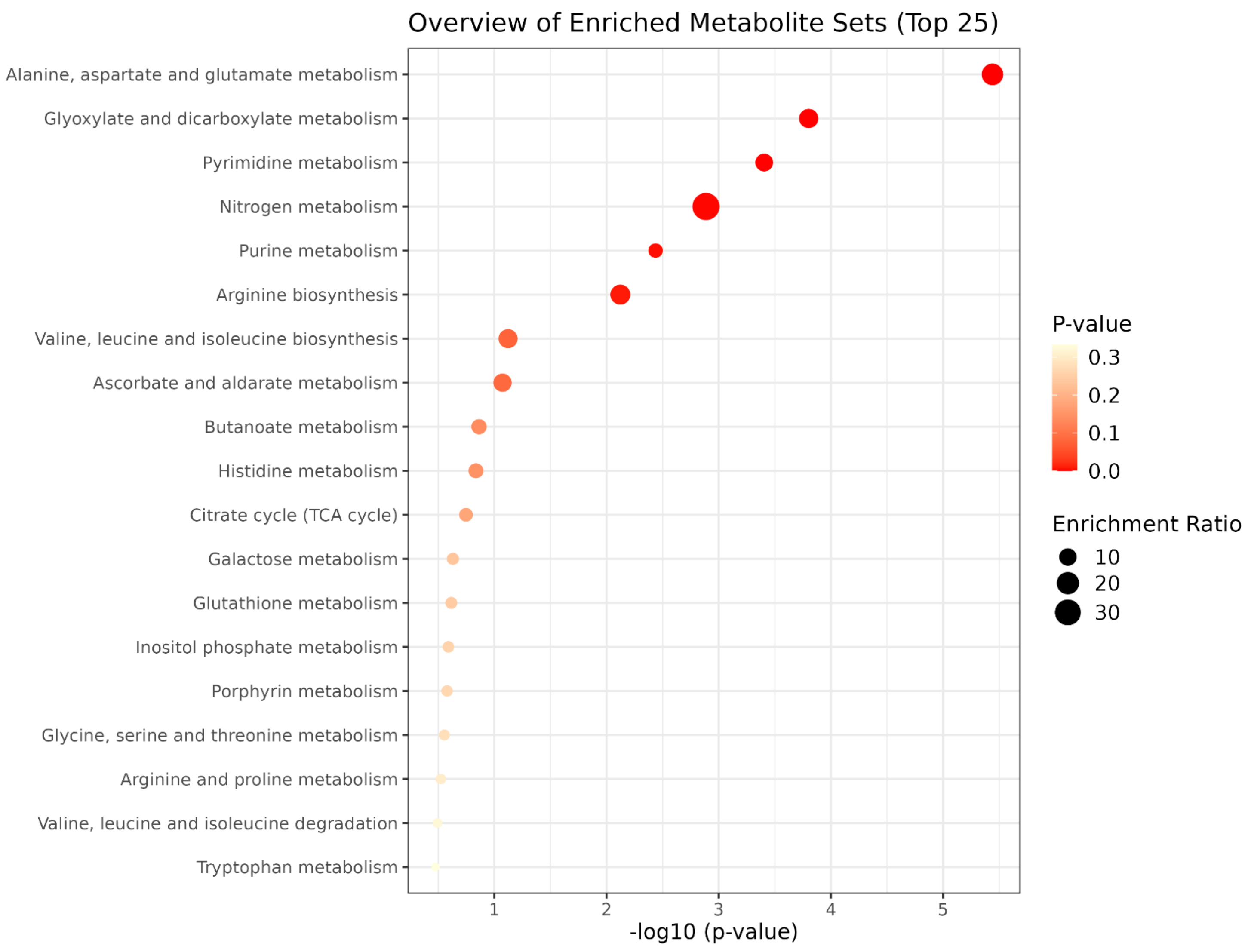

Figure S3: Pathway enrichment analysis of top-ranked metabolites in sea grape extract-treated *H. pylori.*
Metabolic pathway enrichment analysis was performed using the top metabolites contributing to components 1 and 2 in the multivariate model. The analysis was conducted on the MetaboAnalyst 6.0 platform using the KEGG pathway database. The y-axis lists the top 25 significantly enriched pathways, while the x-axis shows the statistical significance ($-\log_{10}$ *p*-value). Dot size represents the enrichment ratio (observed/expected hits), and the color gradient reflects *p*-value, with deeper red indicating higher statistical significance.
